# Acetic acid-indigo carmine chromoendoscopy for delineating early gastric cancers: its usefulness according to histological type

**DOI:** 10.1186/1471-230X-10-97

**Published:** 2010-08-23

**Authors:** Bong Eun Lee, Gwang Ha Kim, Do Youn Park, Dae Hwan Kim, Tae Yong Jeon, Su Bum Park, Hyun Seok You, Dong Yup Ryu, Dong Uk Kim, Geun Am Song

**Affiliations:** 1 Department of Internal Medicine, Pusan National University School of Medicine and Medical Research Institute, Pusan National University Hospital, Busan, Korea; 2 Department of Pathology, Pusan National University School of Medicine, Busan, Korea; 3 Department of Surgery, Pusan National University School of Medicine, Busan, Korea

## Abstract

**Background:**

Endoscopic treatments, such as endoscopic submucosal dissection (ESD) and laparoscopic gastrectomy, are increasingly used to treat a subset of patients with early gastric cancer (EGC). To achieve successful outcomes, it is very important to accurately determine the lateral extent of the tumor. Therefore, we investigated the diagnostic performance of chromoendoscopy using indigo carmine dye added to acetic acid (AI chromoendoscopy) in delineating differentiated or undifferentiated adenocarcinomas in patients with EGC.

**Methods:**

We prospectively included 151 lesions of 141 patients that had an endoscopic diagnosis of EGC. All the lesions were examined by conventional endoscopy and AI chromoendoscopy before ESD or laparoscopic gastrectomy. The border clarification between the lesion and the normal mucosa was classified as distinct or indistinct before and after AI chromoendoscopy.

**Results:**

The borders of the lesions were distinct in 66.9% (101/151) with conventional endoscopy and in 84.1% (127/151) with AI chromoendoscopy (*P *< 0.001). Compared with conventional endoscopy, AI chromoendoscopy clarified the border in a significantly higher percentage of differentiated adenocarcinomas (74/108 [68.5%] vs 97/108 [89.8%], respectively, *P *< 0.001). However, the border clarification rate for undifferentiated adenocarcinomas did not differ between conventional endoscopy and AI chromoendoscopy (27/43 [62.8%] vs 30/43 [70.0%], respectively, *P *= 0.494).

**Conclusions:**

AI chromoendoscopy is useful in determining the lateral extent of EGCs. However, its usefulness is reduced in undifferentiated adenocarcinomas.

## Background

There has been a reduced incidence of gastric cancer in western countries over the past few decades. However, gastric cancer is still the second leading cause of cancer deaths in the world, and it is the most prevalent malignancy in Korea [1,2]. Early gastric cancer (EGC) is defined as a gastric cancer that is confined to the mucosa or submucosa, regardless of the presence or absence of lymph node metastasis [3]. The proportion of EGC cases is increasing in Korea because endoscopic screening for gastric cancer has been adopted [4]. As a result, endoscopic treatments such as endoscopic submucosal dissection (ESD) and laparoscopic gastrectomy are increasingly used to treat a subset of patients with EGC in both Korea and Japan [5-8].

To achieve a successful outcome, it is very important to accurately determine the lateral extent of the tumor. This has traditionally been done with conventional endoscopy and chromoendoscopy using indigo carmine dye [9,10]. However, it is sometimes difficult to identify the margins of the tumors, especially those of superficial or flat-type tumors. Magnifying endoscopes have reportedly been useful in overcoming this difficulty [11,12] but their use is limited by the technical difficulties in manipulating the scopes. Therefore, easier methods are required that make it possible to accurately determine the lateral extent of these tumors. Chromoendoscopy with indigo carmine dye added to acetic acid (AI chromoendoscopy) has recently been reported to improve the diagnostic yield in terms of recognizing the tumor borders in patients with EGC [13,14]. However, the majority of subjects included in these studies had differentiated adenocarcinomas. Therefore, the current study was performed to prospectively investigate the diagnostic performance of AI chromoendoscopy in delineating differentiated or undifferentiated adenocarcinomas in patients with EGC.

## Methods

From January 2007 to May 2009, a total of 151 lesions in 141 patients (85 men and 56 women; age range, 35-81 years; mean age 60 years) with an endoscopic diagnosis of EGC were enrolled prospectively. These patients had previously undergone endoscopic ultrasonography and computed tomography assessments, and were scheduled to undergo ESD or surgery.

This study was approved by the Institutional Review Board at Pusan National University Hospital and informed consent was obtained from all the patients before their examination.

### Diagnostic procedures

**Figure 1:**
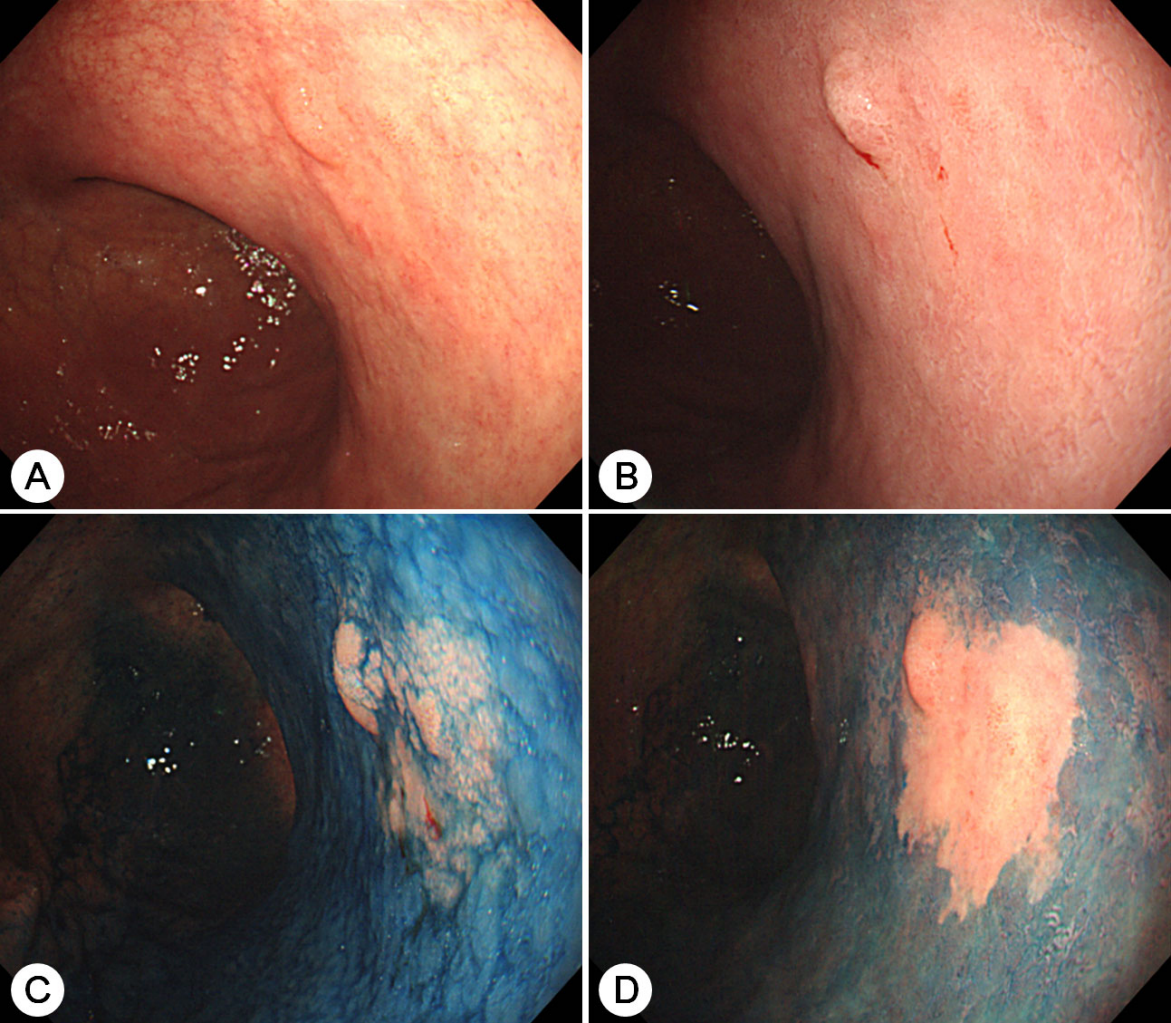
**Chromoendoscopy of a differentiated adenocarcinoma**. (A) A combined flat and elevated lesion with an unclear border at the lower body of the stomach is shown. (B) Endoscopic view after acetic acid was sprinkled. (C) Endoscopic view after indigo carmine was additionally sprinkled. (D) Endoscopic view after the lesion was washed with clean water. After chromoendoscopy with indigo carmine dye added to acetic acid, the lesion's borders became distinct and the clarity of the image is high. The lesion was resected by endoscopic submucosal dissection and was shown to be a differentiated adenocarcinoma.

**Figure 2:**
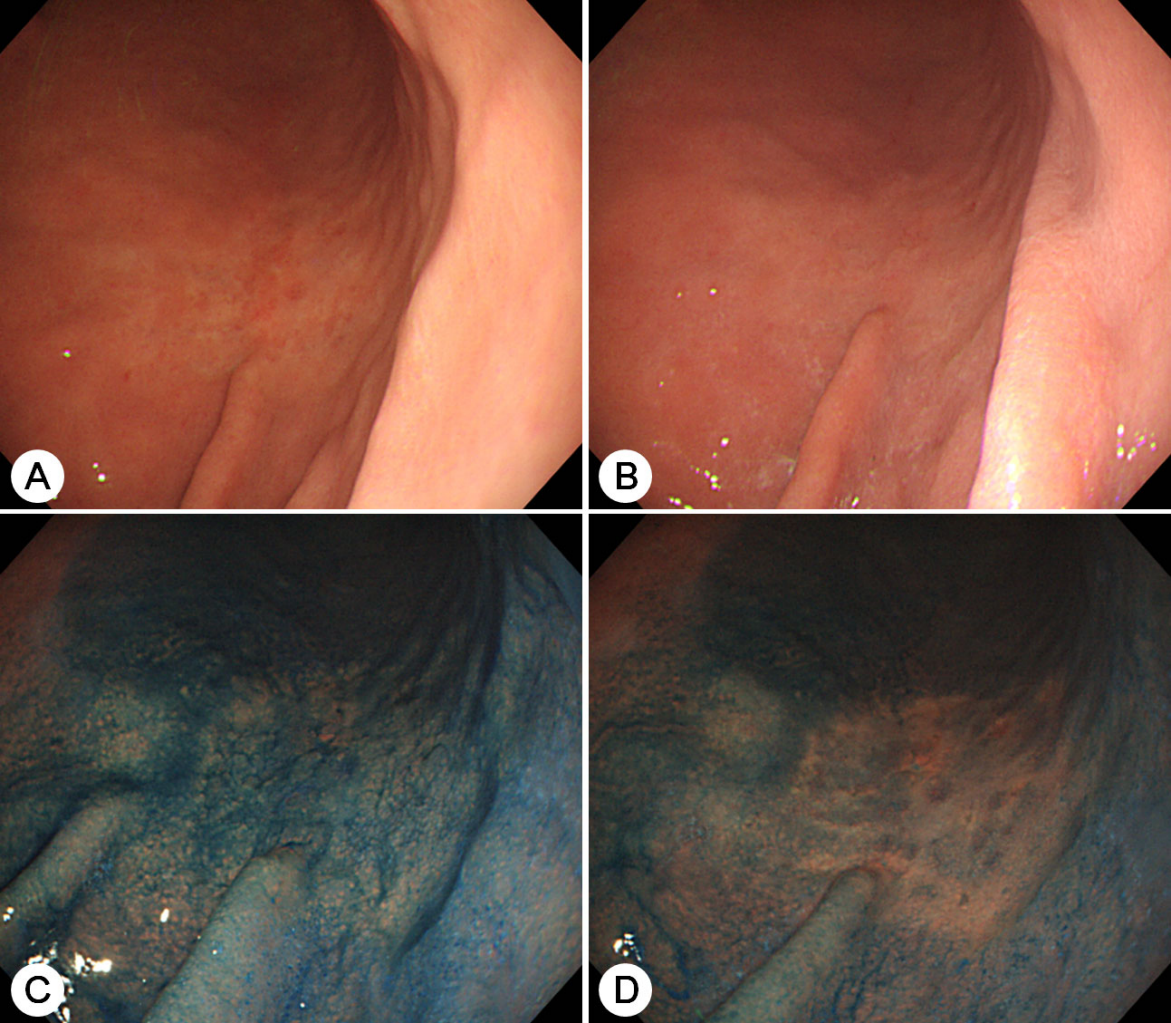
**Chromoendoscopy of an undifferentiated adenocarcinoma**. (A) A flat discolored lesion with an unclear border at the lower body of the stomach is shown. (B) Endoscopic view after acetic acid was sprinkled. (C) Endoscopic view after indigo carmine was additionally sprinkled. (D) Endoscopic view after the lesion was washed with clean water. After chromoendoscopy with indigo carmine dye added to acetic acid, the lesion's border was still indistinct and the image was mottled. The lesion was resected by laparoscopic gastrectomy and was shown to be an undifferentiated adenocarcinoma.

All the lesions detected by high-definition video endoscopy (EVIS LUCERA GIF-H260; Olympus Optical Co., Ltd, Tokyo, Japan) were examined by a single experienced endoscopist (G.H. Kim) as follows: step 1, mucus adhering to the mucosa was washed away as thoroughly as possible before the examination of the lesion; step 2, 10-20 mL of 1.5% acetic acid was sprinkled evenly over and around the lesion using a washing pipe (PW-5L-1; Olympus); step 3, 10-20 mL of 0.2% indigo carmine dye was similarly sprinkled 30-60 seconds later using a washing pipe; step 4, the area was washed with clean water 20-30 seconds later for the final view (Figure 1, 2).

Before and after AI chromoendoscopy, the border clarification between the lesion and the normal mucosa was classified as distinct or indistinct by observation with the naked eye. The clarity of the endoscopic image after AI chromoendoscopy was also classified as clear, mottled, or unclear.

### Clinicopathological review

ESD or laparoscopic gastrectomy was performed within one week of AI chromoendoscopy. The resected specimens were fixed in 10% buffered formalin. Carcinomas with adjacent non-neoplastic mucosa were serially cut into 2 mm parallel slices and embedded in paraffin, and then sectioned and stained with hematoxylin-eosin for histological examination. The clinicopathological findings, such as age, sex, macroscopic shape, tumor site, tumor size, histological type and depth of invasion, were reviewed according to the Japanese Classification of Gastric Carcinomas [15]. The depth of tumor invasion was classified as mucosal, submucosal, or advanced (the tumor had invaded the muscularis propria or deeper).

### Statistical analysis

The difference in the border clarification when conventional endoscopy or AI chromoendoscopy was used and the differences in the clinicopathological characteristics according to the border clarification and the clarity of the endoscopic image were assessed using χ^2 ^test or Fisher's exact test. Multivariate logistic regression analysis was used to identify variables predicting the border clarification after AI chromoendoscopy. Influencing factors/covariates for the border clarification were tumor size, macroscopic type, histopathological diagnosis and depth of tumor invasion. A *P *value < 0.05 was considered statistically significant. The statistical calculations were performed with the SPSS version 12.0 for Windows software (SPSS Inc., Chicago, IL, USA).

## Results

The clinicopathological characteristics of the patients enrolled in this study are summarized in Table 1. Sixty lesions were treated by ESD and 91 lesions were treated by laparoscopic gastrectomy. The lesions were located in the upper third of the stomach for 7.3% (11/151), in the middle third for 39.7% (60/151) and in the lower third for 53.0% (80/151). Macroscopically, the lesions were classified as flat/elevated (64/151, 42.4%), depressed (64/151, 42.4%), or excavated (23/151, 15.2%). Histopathologically, the lesions were diagnosed as differentiated adenocarcinomas in 71.5% (108/151) and undifferentiated adenocarcinomas in 28.5% (43/151). The depth of tumor invasion was mucosal in 71.5% (108/151), submucosal in 25.8% (39/151), and advanced in 2.7% (4/151).

**Table 1:** Clinicopathological characteristics of the patients

Patients	141
Sex (male/female)	85/56
Mean age (year)	60 (range 35-81)
Tumors	151
Mean tumor size (mm)	22 (range 3-88)
Location	
Upper third of the stomach	11
Middle third of the stomach	60
Lower third of the stomach	80
Macroscopic type	
Flat/Elevated	64
Depressed	64
Excavated	23
Concomitant ulceration	
Present	35
Absent	116
Histopathological diagnosis	
Differentiated	108
Undifferentiated	43
Depth	
Mucosal	108
Submucosal	39
Advanced	4

**Figure 3:**
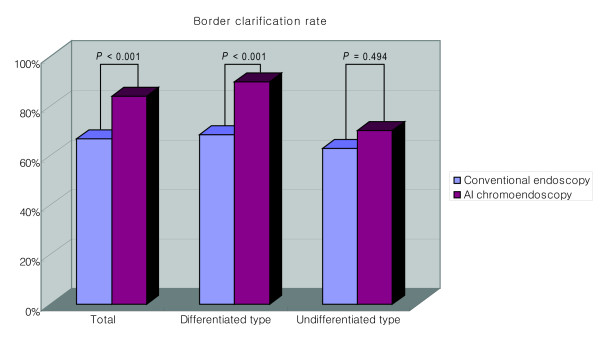
**The rates of border clarification by conventional endoscopy and chromoendoscopy with indigo carmine dye added to acetic acid (AI chromoendoscopy) according to the histological type of the lesion**.

The border of the lesion was distinct in 66.9% (101/151) with conventional endoscopy and in 84.1% (127/151) with AI chromoendoscopy (*P *< 0.001) (Table 2) (Figure 3). Compared with conventional endoscopy, AI chromoendoscopy clarified the border in a significantly higher percentage of differentiated adenocarcinomas (74/108 [68.5%] vs 97/108 [89.8%], respectively, *P *< 0.001). However, the border clarification rate for undifferentiated adenocarcinomas did not differ between conventional endoscopy and AI chromoendoscopy (27/43 [62.8%] vs 30/43 [70.0%], respectively, *P *= 0.494). Of the 50 lesions with indistinct borders on conventional endoscopy, AI chromoendoscopy clarified the borders in 66.0% (33/50) (27 of 34 differentiated adenocarcinomas and 6 of 16 undifferentiated adenocarcinomas).

**Table 2:** Border clarification of the lesions before and after chromoendoscopy with indigo carmine dye added to acetic acid

	After chromoendoscopy					
	
	Total (n = 151)		Differentiated adenocarcinomas (n = 108)		Undifferentiated adenocarcinomas (n = 43)	
	Distinct margin	Indistinct margin	Distinct margin	Indistinct margin	Distinct margin	Indistinct margin
Conventional endoscopy						
Distinct margin	94	7	70	4	24	3
Indistinct margin	33	17	27	7	6	10

When the indistinct border group was compared with the distinct border group after AI chromoendoscopy, there were no differences in the tumor size, location, or depth (Table 3). The lesions with an excavated morphology showed a higher frequency of indistinct borders than those with a flat/elevated or depressed morphology (*P *= 0.034). Undifferentiated carcinomas had a higher frequency of indistinct borders than differentiated carcinomas (13/43 [30.2%] vs 11/108 [10.2%], respectively, *P *= 0.002). On multivariate regression analysis, only the histopathological type was associated with the border clarification (Table 4).

**Table 3:** Clinicopathological characteristics of the lesions of the distinct border group and the indistinct border group after chromoendoscopy with indigo carmine dye added to acetic acid

	Distinct border group (n = 127)	Indistinct border group (n = 24)	*P *value
Tumor size			0.232
≤ 2 cm	80	12	
> 2 cm	47	12	
Location			0.788
Upper third of the stomach	10	1	
Middle third of the stomach	51	9	
Lower third of the stomach	66	14	
Macroscopic type			0.034
Flat/Elevated	59	5	
Depressed	52	12	
Excavated	16	7	
Histopathological diagnosis			0.002
Differentiated	97	11	
Undifferentiated	30	13	
Depth			0.273
Mucosal	93	15	
Submucosal	30	9	
Advanced	4	0	

**Table 4:** Multivariate analysis for the border clarification after chromoendoscopy with indigo carmine dye added to acetic acid

Variables	Coefficient	SE	OR (95% CI)	*P *value
Tumor size > 2 cm	-0.269	0.473	0.76 (0.30-1.93)	0.569
Excavated morphology	-0.688	0.555	0.50 (0.17-1.49)	0.215
Undifferentiated carcinoma	-1.143	0.485	0.32 (0.12-0.83)	0.018
Submucosal/advanced invasion	-0.190	0.496	0.83 (0.31-2.18)	0.701

SE: standard error; OR: odds ratio; CI: confidence interval.

The clarity of the lesions after AI chromoendoscopy did not differ according to the tumor size, location, or depth (Table 5). The lesions with a flat/elevated morphology showed a higher frequency of clear images than those with a depressed or excavated morphology (*P *= 0.009). Undifferentiated adenocarcinomas showed a higher frequency of mottled appearance than differentiated adenocarcinomas (30/43 [69.8%] vs 2/108 [1.9%], respectively, *P *= 0.002).

**Table 5:** Clinicopathological characteristics of the lesions according to the clarity of the endoscopic image after chromoendoscopy with indigo carmine dye added to acetic acid

	Endoscopic image after chromoendoscopy			*P *value
		
	Clear (n = 83)	Mottled (n = 32)	Unclear (n = 36)	
Tumor size				0.381
≤ 2 cm	50	17	25	
> 2 cm	33	15	11	
Location				0.140
Upper third of the stomach	7	3	1	
Middle third of the stomach	29	18	13	
Lower third of the stomach	47	11	22	
Macroscopic type				0.009
Flat/Elevated	45	8	11	
Depressed	30	15	19	
Excavated	8	9	6	
Histopathological diagnosis				0.002
Differentiated	77	2	29	
Undifferentiated	6	30	7	
Depth				0.222
Mucosal	63	19	26	
Submucosal	17	13	9	
Advanced	3	0	1	

## Discussion

In this study, the diagnostic performance of conventional endoscopy in recognizing tumor borders was inadequate (only 66.9%) in patients with an endoscopic diagnosis of EGC. AI chromoendoscopy increased the recognition rate of tumor borders to 84.1%, especially in differentiated adenocarcinomas.

The accurate determination of the pre-treatment lateral extent of a tumor is critical for successful endoscopic resection and laparoscopic gastrectomy in patients with EGC. Inadequate determination of the lateral extent may result in an incomplete resection, which would increase the rate of local relapse. During endoscopic resection such as ESD, the entire border between the tumor and the normal mucosa is electrosurgically marked, approximately 5 mm from the lesion, and the procedure is then performed [16]. During a surgical operation such as a laparoscopic gastrectomy, EGC lesions cannot be identified by inspecting the serosal surface and are usually impossible to palpate manually because the depth of the invasion is shallow. Therefore, the day before surgery, two or three endoscopic clips are usually placed at the mucosa approximately 1-2 cm from the proximal margin of the lesion in the oral direction [17]. In this study, we used the same localization technique for endoscopic resection and laparoscopic gastrectomy.

The diagnostic performance for determining the lateral extent of a tumor with conventional endoscopy is inadequate because the tumor rims are often almost the same height and color as the surrounding normal mucosa [13]. Chromoendoscopy with indigo carmine dye, which is not absorbed by the mucosa but pools in crevices and valleys, thus defining the irregularities in the mucosal architecture, has been used for over 30 years and is still a strong modality for identifying gastric lesions [9,10,13]. However, the accurate delineation of the tumor area is often difficult because the dye simply contrasts the surface irregularity of the tumor [18].

Magnifying endoscopy has recently been reported as useful in determining the lateral spread of gastric cancers [11,12]. However, magnifying endoscopy is not popular and there is no generally accepted standard for identifying the patterns of tumors, which limits the role of magnifying endoscopy in determining the lateral extent of a tumor.

The technique based on the application of acetic acid during the endoscopy was first used to observe the specialized columnar epithelium of Barrett's esophagus [19]. This technique was then adopted for the assessment of gastric neoplasms [11,20]. The transient whitish colorization of the epithelial surface, which occurs after the spraying of acetic acid, is a consequence of the increased opacity. This corresponds to a reversible alternation of the three-dimensional structures of the cytoplasmic proteins [13]. However, the lateral margins were successfully identified with acetic acid in only 42-53% of gastric neoplasms [13].

Based on chromoendoscopy with acetic acid, Yamashita et al. recently described the use of an indigo carmine and acetic acid mixture to accurately identify the margins of gastric cancers in 27 cases, which was even possible with low-resolution endoscopy [18]. The specificity and sensitivity were 98.0% and 100%, respectively, based on biopsy samples from the demarcated areas or just outside the areas. In a prospective study of 53 neoplasms, which compared AI chromoendoscopy with conventional chromoendoscopy using indigo carmine or acetic acid alone, the diagnostic performance of AI chromoendoscopy (94.3%) was significantly better than that of the other modalities [13]. Of the 53 lesions, 49 were differentiated adenocarcinomas, 3 were adenomas and only one lesion was an undifferentiated adenocarcinoma. Similarly, in our present study, AI chromoendoscopy clarified the borders in 89.8% (97/108) of the differentiated adenocarcinomas.

However, there has been no report on the performance of AI chromoendoscopy in the assessment of undifferentiated adenocarcinomas. In the present study, the borders of undifferentiated adenocarcinomas were distinct in 62.8% with conventional endoscopy and in 70.0% with AI chromoendoscopy. Of the 16 undifferentiated adenocarcinomas with an indistinct border during conventional endoscopy, AI chromoendoscopy clarified the borders in only 6 lesions. Therefore, the diagnostic performance of AI chromoendoscopy in assessing undifferentiated adenocarcinomas seems to be unsatisfactory compared with the diagnostic performance of AI chromoendoscopy in assessing differentiated adenocarcinomas.

We also investigated the differences in the clinicopathological characteristics of the lesions in the distinct border group and the indistinct border group after AI chromoendoscopy. The frequency of an indistinct border was higher for lesions with an excavated morphology as well as with undifferentiated adenocarcinoma. During this study, we discovered that there was a difference between differentiated and undifferentiated adenocarcinomas in the clarity of the lesions after AI chromoendoscopy. A mottled appearance was more common in undifferentiated adenocarcinomas than in differentiated adenocarcinomas. These results can be explained by the fact that undifferentiated adenocarcinomas infiltrate diffusely among the normal gastric glandular cells.

The exact mechanism of AI chromoendoscopy is still unclear, but we propose a possible mechanism. When acetic acid is sprinkled, the surrounding non-cancerous mucosa whitens, but the cancerous mucosa does not, which produces good contrast between the pinkish cancer lesion and the surrounding non-cancerous tissue. If indigo carmine is additionally sprinkled, then the surrounding whitish non-cancerous mucosa is stained blue and the pinkish cancer is not stained. This color difference is made clearer by washing with clean water.

However, in seven of the 101 lesions with clear borders on conventional endoscopy, the borders became less clear after AI chromoendoscopy. This problem would be attributed to the increased secretion of mucus from the gastric mucosa after the acetic acid was sprinkled on it, which resulted in the adhesion of the mucus to the surface of the lesion, reducing the contrast between the lesion and the normal mucosa [14].

## Conclusions

AI chromoendoscopy is useful in determining the lateral extent of EGCs. However, the usefulness of AI chromoendoscopy is reduced in lesions with an excavated morphology and in undifferentiated adenocarcinomas.

## Competing interests

The authors declare that they have no competing interests.

## Authors' contributions

GHK, DYP and GAS conceived and designed the study. BEL, SBP, HSY and DYR analyzed the data. BEL and GHK drafted the manuscript, and DYP, DHK, TYJ and DUK revised it. All authors read and approved the final manuscript.

## Pre-publication history

The pre-publication history for this paper can be accessed here:

http://www.biomedcentral.com/1471-230X/10/97/prepub
